# Clinical patterns in CADASIL

**DOI:** 10.1007/s00415-022-11261-1

**Published:** 2022-07-14

**Authors:** G. Gailani, N. P. Robertson

**Affiliations:** 1 Department of Neurology, University Hospital of Wales, Heath Park, Cardiff, CF14 4XN UK; 2 Division of Psychological Medicine and Clinical Neuroscience, Department of Neurology, Cardiff University, University Hospital of Wales, Heath Park, Cardiff, CF14 4XN UK

## Introduction

Cerebral autosomal dominant arteriopathy with subcortical infarcts and leukoencephalopathy (CADASIL) is the most common hereditary cause of small vessel disease. It commonly carries a poor prognosis with affected individuals developing cognitive and neuroimaging features by the age of 45–55. The genetic basis for the disorder is highly stereotyped mutations within the extracellular domain of the NOTCH3 receptor [Notch3(ECD)] on chromosome 19 that result in an odd number of cysteine residues within the epidermal growth factor-like repeat domain. Advances in genetics now raise the possibility of more effective interventions. However, a better understanding of genetic variation and disease evolution together with the development of outcome measures are required.

In journal club this month, we review three papers exploring these issues. The first paper explores clinical expression of NOTCH*3*^cys^ variants within EGFr (epidermal growth-factor-like repeat) domains. The second study investigates the use of Optical coherence tomography angiography (OCT-A), a non-invasive technique used to visualise retinal vasculature at high resolution, to determine the correlation between the macular vessel density and inner retinal thickness and the severity of CADASIL. The final paper determines the prevalence of vascular cognitive impairment (VCI) in a cohort of CADASIL patients, and explores factors associated with VCI risk.

## Family members with a pathogenic *NOTCH3* variant can have a normal brain magnetic resonance imaging and skin biopsy beyond age 50 years

Previous studies have demonstrated that clinical expression of NOTCH-3 mutations in the general population is highly variable between individuals and families, ranging from asymptomatic mild small vessel disease to more severe forms of dementia and cerebral vascular disease. The reasons for this are not well established, although vascular risk factors have been associated with more rapid progression. Extremely mild NOTCH-3-associated small vessel disease (mSVD^NOTCH3^) phenotypes in CADASIL pedigrees have not previously been documented.

Remco et al. identified 7 individuals (mean age 56.6) from the cross-sectional Dutch CADASIL pedigree cohort (*n* = 200) fulfilling predetermined criteria for mSVD^NOTCH3^,and explored differences in MR brain imaging as well as skin biopsy immunohistochemistry and electron microscopy characteristics. All seven patients had a *NOTCH3*^cys^ variant in one of EGFr domains 7 to 34: 6 had a variant in EGFr domain 14, one individual had a variant in EGFr domain 17. Brain MRI of mSVD^*NOTCH3*^ cases were quantitatively assessed for SVD imaging markers and compared with brain MRIs of first-degree relatives with a *NOTCH3*^cys^ variant as well as the 89 patients with an EGFr 7–34 variant from the Dutch CADASIL cohort. All but one of the mSVD^NOTCH3^ individuals had normal MR brain imaging. In addition, mSVD^NOTCH3^ cases had only very low levels of NOTCH3 ectodomain aggregation which was significantly less than in symptomatic EGFr 7–34 CADASIL patients.

*Comment*: This study demonstrates that extremely mild SVD phenotypes can occur in individuals from CADASIL pedigrees with *NOTCH3*^cys^ EGFr 7–34 variants with normal brain imaging up to the age of 58. This has relevance in clinical practice, as presence of the mutation does not mean that a carrier will inevitably develop the more severe classical CADASIL phenotype.

Ramco et al. Stroke. 2022;53(6):1964–1974. https://doi.org/10.1161/STROKEAHA.121.036307

## Reduced macular vessel density and inner retinal thickness correlate with the severity of CADASIL

Retinal and cerebral arterioles share similar embryonic, anatomic, and physiological features. Deposition of granular osmiophilic material (GOM) in the smooth muscle cells of vessel walls is the pathological signature of the arteriopathy in CADASIL and have previously been reported in retinal vessels together with vessel wall thickening, sheathing, and narrowing of retinal arterioles, retinal nerve fibre loss and cotton wool spots. This case control study investigated the correlation between OCT-A and cognitive, gait function, and MRI markers in patients with CADASIL making comparison with healthy controls.

Thirty-five patients with clinical and neuroimaging features of cerebral SVD and a confirmed NOTCH3 CADASIL and 35 healthy controls age-matched controls without diabetes, vascular disease, dementia or other neurological or psychiatric disease were enrolled in the study. The CADASIL group was further subdivided into stroke (focal neurological dysfunction for > 24 h with corresponding evidence of cerebral infarction of haemorrhage) and non-stroke groups. Participants then underwent detailed MRI brain, visual assessment, OCT-A, cognitive and gait assessment. Patients or controls with evidence of disorders that could potentially result in abnormal OCT-A results such as diabetic/hypertensive retinopathy or age-related macular degeneration were excluded.

Analyses were conducted on 59 eyes of 35 patients with CADASIL and 54 eyes of 35 controls. The CADASIL stroke and non-stroke group comprised 20 and 15 individuals, respectively. OCT-A parameters between cases and controls were comparable. However, the OCT-A the macular vessel density in the superficial retinal plexus was significantly lower in the CADASIL stroke subgroup than in the non-stroke subgroup, as was the inner retinal thickness, although this did not correlate with burden of WMH on MRI. Macular vessel density and inner retinal thickness were positively correlated with gait speed and negatively correlated with number of lacunae.

*Comment*: The study had several limitations, first, the small number of participants, which makes it difficult to generalise results. Second, the correlation in retinal vessel density and functional status found in the CADASIL stroke group may be difficult to interpret as this group tended to be older and had more cardiovascular risk factors than the non-stroke group. It has also been established from previous studies that the extent of WMH on MRI does not correlate well with the severity of CADASIL. However, this study does offer the potential for a non-invasive tool to measure outcomes in CADASIL although larger studies will be required to validate these initial findings.

Lin et al. PLoS One 17(5): e0268572. https://doi.org/10.1371/journal.pone.0268572

## Prevalence and predictors of vascular cognitive impairment in patients with CADASIL

This case control study recruited CADASIL patients from six centres in the UK as part of a national prospective study of familial cerebral small vessel disease and 265 healthy age- and sex-matched controls from a previous Brief Memory and Executive Test (BMET) validation study. For CADASIL cases, information had been collected prospectively on clinical presentation, vascular risk factors and family history, and original clinical brain MRI images were also available. The first 265 CADASIL patients recruited to the national study were included of which 176 had cognitive assessments available and were included in analysis. All patients had typical cysteine changing mutations. Cognition was assessed using the BMET and Montreal Cognitive Assessment (MoCA) and correlated vascular cognitive impairment (VCI) with clinical, genetic, and imaging characteristics.

Unsurprisingly, VCI was more prevalent in cases than controls; 39.8 vs 10.2% on BMET and 47.7 vs 19.6% on MoCA and CADASIL cases performed worse across all cognitive domains with the most prominent deficits observed in executive function and processing speed. In addition, a prior history of stroke was associated with VCI after controlling for age and sex but there was no association of VCI with site of mutation. Overall, MRI parameters showed a poor correlation with VCI with no differences in white matter hyperintensities lesion volume, cerebral microbleed count and normalised brain volume between those with and without VCI on either the BMET or MoCA. Lacune count was the only MRI parameter independently associated with VCI on the BMET.

*Comment*: The study is a well-constructed study of VCI in CADASIL and reveals that at a mean age of 51, half of the participants in the CADASIL group had VCI and that the most consistent risk factor was prior stroke. The authors also suggest that the BMET is superior to detect VCI in CADASIL patients as it is primarily designed to be particularly sensitive to impairment in executive function and processing speed seen in SVD, whilst the MoCA was developed as a global cognitive score primarily to assess cortical dementias. Further expansion of this cohort and continued collection of prospective data are likely to provide further valuable insights.

Jolly et al. Neurology. 2022 May 23:10.1212/WNL.0000000000200607. https://doi.org/10.1212/WNL.0000000000200607

